# Retraction: High performance flexible supercapacitors based on secondary doped PEDOT–PSS–graphene nanocomposite films for large area solid state devices

**DOI:** 10.1039/d5ra90034d

**Published:** 2025-04-07

**Authors:** Syed Khasim, Apsar Pasha, Nacer Badi, Mohana Lakshmi, Yogendra Kumar Mishra

**Affiliations:** a Department of Physics, Faculty of Science, University of Tabuk Tabuk 71491 Kingdom of Saudi Arabia syed.pes@gmail.com; b Renewable Energy Laboratory, Nanotechnology Research Unit, Faculty of Science, University of Tabuk Tabuk 71491 Kingdom of Saudi Arabia; c Department of Physics, PES University Bangalore 560100 Karnataka India; d Department of Physics, Gousia College of Engineering Ramanagaram 562159 Karnataka India; e Mads Clausen Institute, Nano SYD, University of Southern Denmark Alsion 2 6400 Sønderborg Denmark

## Abstract

Retraction of ‘High performance flexible supercapacitors based on secondary doped PEDOT–PSS–graphene nanocomposite films for large area solid state devices’ by Syed Khasim *et al.*, *RSC Adv.*, 2020, **10**, 10526–10539, https://doi.org/10.1039/D0RA01116A.

The Royal Society of Chemistry hereby wholly retracts this *RSC Advances* article due to concerns with the reliability of the data.

The photographic images of a thin film and an LED presented in Fig. 2 have been reproduced from previous articles by different authors and used without permission.^1,2^ The authors have not been able to satisfactorily explain why they did not use images of their own free-standing films or LED in Fig. 2, nor provide any photographic evidence to prove that they did produce their own films.

In addition, the cyclic voltammetry data reported in Fig. 7a–c appear to contain duplicated datasets. The authors provided raw data, but upon analysis by an expert, it was concluded that the raw data was not authentic and showed evidence of manipulation.

Given the significance of these concerns, the findings presented in this paper are no longer reliable.

This retraction supersedes the information provided in the Expression of concern related to this article.

Syed Khasim, Nacer Badi and Yogendra Kumar Mishra agree to the retraction. The authors have offered to repeat the analysis, but the Royal Society of Chemistry does not feel this is appropriate due to the concerns regarding the reliability of the original data. The other authors did not respond.

Signed: Laura Fisher, Executive Editor, *RSC Advances*

Date: 25th March 2025
